# Alternative molecular and genomic strategies to provide a rapid response to alerts concerning the introduction of new emerging SARS-CoV-2 variants: the Omicron alert

**DOI:** 10.1128/spectrum.01075-23

**Published:** 2023-09-22

**Authors:** Marta Herranz, Sergio Buenestado-Serrano, Javier Martín-Escolano, Andrea Molero-Salinas, Roberto Alonso, Pilar Catalán, Patricia Muñoz, Darío García de Viedma, Laura Pérez-Lago, Gregorio Marañón Microbiology-ID COVID 19 Study Group, Luis Alcalá

**Affiliations:** 1 Servicio de Microbiología Clínica y Enfermedades Infecciosas, Gregorio Marañón General University Hospital, Madrid, Spain; 2 Instituto de Investigación Sanitaria Gregorio Marañón (IiSGM), Madrid, Spain; 3 Escuela de Doctorado, Universidad de Alcalá, Alcalá de Henares, Madrid, Spain; 4 CIBER Enfermedades Respiratorias (CIBERES), Madrid, Spain; 5 Departamento de Medicina, Universidad Complutense, Madrid, Spain; 6 CIBER Enfermedades Infecciosas (CIBERINFEC), Madrid, Spain; Emory University School of Medicine, Atlanta, Georgia, USA

**Keywords:** SARS-CoV-2, Omicron, genomic strategies, molecular tools, VOC alert

## Abstract

During the COVID-19 pandemic, several SARS-CoV-2 variants of concern (VOCs) of particular relevance emerged. Early detection of VOCs entering a country is essential to control spread. The alert triggered by the first suspected case of the Omicron variant in Spain in a traveler arriving from South Africa in November 2021 provided a unique opportunity to evaluate four different methodological strategies tailored to rapid identification of Omicron. The different approaches were designed to respond to the different technical resources available in different settings. First, we used melting probes in RT-PCR to determine the presence of four Omicron signatures (K417N, E484A, P681H, and absence of L452R): three probes showed deviations in temperature (Tm) values relative to the reference codons (E484K-15.8°C, P681H-5.2°C, and L452R-7.2°C) and one maintained the reference value (K417N). The deviation in Tm of P681H suggested the presence of the characteristic Omicron N679K mutation in the probe hybridization region; these data pointed to the presence of Omicron alleles. Second, the presence of 29 of the 33 characteristic single nucleotide polymorphisms (SNPs) in the Omicron variant S-gene was identified by Sanger sequencing of nine amplicons. The final two strategies involved identification of 47 of the 50 non-synonymous and indel mutations attributed to Omicron by rapid nanopore whole genome sequencing (WGS) and by Illumina WGS technology. These strategies enabled us to pre-assign the first Omicron case in Spain with high certainty 2 h after receipt of RNA and to confirm it genomically 3 h later, so that the Public Health authorities could be rapidly notified.

**IMPORTANCE:**

The study presents different experimental alternatives to identify new variants of concern (VOCs) of SARS-CoV-2 entering a certain population. Early detection of a new VOC is crucial for surveillance and control of spread. The objective is to provide laboratories with tools adapted to their resource capabilities that offer a sufficient level of resolution to rule out, confirm, or pre-assign the presence of a suspected VOC. The study describes four different techniques that were applied simultaneously to the first suspected Omicron case in Spain, highlighting the level of resolution and response time achieved in each case. These techniques are based on the detection of mutations in the S-gene of the virus that can easily adapt to potential emerging variants. The results of the study allow any laboratory to prepare for new alerts of SARS-CoV-2 VOCs.

## INTRODUCTION

Since the outbreak of SARS-CoV-2 was declared a global pandemic on 11 March 2020, we have witnessed the constant evolution of the virus and the appearance of multiple variants, some of which have been designated variants of concern (VOCs) because of their impact on public health. According to the World Health Organization (WHO), a variant of concern (VOC) of SARS-CoV-2 is a virus variant that has been shown to be associated with (i) an increase in transmissibility or a detrimental change in COVID-19 epidemiology, (ii) an increase in virulence or a change in clinical disease presentation, or (iii) a decrease in the effectiveness of public health and social measures or available diagnostics, vaccines, or therapeutics. To date, five VOCs have been identified: Alpha (B.1.1.7) in September 2020 (UK), Beta (B.1.351) in September 2020 (South Africa), Gamma (P.1) in December 2020 (Brazil), Delta (B.1.617.2) in December 2020 (India), and the latest to be declared was Omicron (B.1.1.529) in November 2021 (South Africa) (1, 2).

Thanks to constant genomic surveillance and analyses being carried out worldwide, new variants can be identified and a global alert issued for further monitoring and in-depth studies (3, 4). Once a potential VOC has been identified, it is important to assess its geographical distribution and try to control its spread until the most appropriate action measures related to the surveillance and isolation of patients and contacts can be determined. Rapid detection of new relevant SARS_CoV_2 variants entering a country for the first time is essential because of the potential public health implications, and diagnostic laboratories must be prepared to identify it accurately in the shortest possible time.

The latest VOC to raise global alarm was Omicron (B.1.1.529), first identified in South Africa and reported to the WHO on 24 November 2021. Since then, Omicron has diversified into 5 lineages and more than 200 sub-lineages, with XBB.1.5, XBB.1.9, and XBB.1.16 currently dominating globally (5, 6). Omicron variants have an unusually high number of mutations compared to the other VOCs described to date, accumulating more than 30 single nucleotide polymorphisms (SNPs) in the S-gene of the SPIKE protein and more than 50 mutations compared to the ancestral SARS_CoV_2 strain (Wuhan-HU-1, NC_045512.2) (7, 8). There is considerable uncertainty and controversy regarding the origin and appearance of this variant. It has been suggested that the origin of this particular variant may have been within-host evolution during infection in one or more patients with persistent infection (9, 10) or that lack of sampling for several months in southern Africa led to the loss of the necessary intermediate mutations (9) or that it could have an animal origin, with variants evolving via a human-cat-mouse-human circular pathway (11).

The aim of this study is to propose four alternative methodological strategies to identify emerging SARS-CoV-2 variants rapidly and accurately. The purpose of this four-pronged strategy is to design methods adapted to laboratories with different resources and analytical requirements in terms of speed of response and accuracy. We conducted this research under the conditions of a real public health alert at the time the health alert notice of the possible first entry of Omicron into Spain was issued.

Two of the strategies tested provided a rapid response on the day of diagnosis: the first aimed to pre-assign the variant based on targeted RT-PCR of a selection of highly informative SNPs; the second provided definitive confirmation by ultra-rapid whole-genome nanopore sequencing using the MinION system (Oxford Nanopore Technology). The other two approaches took 2 and 3 working days; the first, based on Sanger sequencing, targeted a large number of informative SNPs to ensure accurate variant confirmation and the second, using Illumina technology, to obtain whole-genome sequencing data.

## MATERIALS AND METHODS

For identification of the new Omicron variant detected in Spain on 29 November 2021, non-synonymous mutations and indels in the B.1.1.529 variant reported on 24 November 2021 (Table 1) were used.

**TABLE 1:** Results of the detection of non-synonymous and indel mutations described for the Omicron variant (on 29 November 2021) using different sequencing techniques: Oxford Nanopore, Illumina, and Sanger

Gene	SNP/indel	Amino acid substitution	Oxford Nanopore sequencing	Illumina sequencing	Sanger sequencing
ORF1ab	A2832G	K856R	G	G	NA^*b*^
ORF1ab	6513_6515del	del2084/2084	A-[GTT]A	A-[GTT]A	NA
ORF1ab	G8393A	A2710T	A	A	NA
ORF1ab	C10029T	T3255I	T	T	NA
ORF1ab	C10449A	P3395H	A	A	NA
ORF1ab	11288_11296del	del3674/3676	A-[GTTTGTCTG]G	A-[GTTTGTCTG]G	NA
ORF1ab	G11287T	L3674F	T	T	NA
ORF1ab	A11537G	I3758V	G	G	NA
ORF1ab	C14408T	P4715L	T	T	NA
ORF1ab	A18163G	I5967V	G	G	NA
S	C21762T	A67V	T	T	T
S	21765_21770del	del69/70	A-[TACATG]T	A-[TACATG]T	A-[TACATG]T
S	C21846T	T95I	T	T	T
S	G21987A	G142D	A	A	A
S	21987_21995	del143/145	G-[GTGTTTATT]A	G-[GTGTTTATT]A	G-[GTGTTTATT]A
S	22194_22196del	del212/212	A-[ATT]T	A-[ATT]T	NR
S	22205GAGCCAGAAins	215EPEins	GAGCCAGAA	GAGCCAGAA	NR
S	G22578A	G339D	A	A	A
S	T22673C, C22674T	S371L	CT	CT	CT
S	T22679C	S373P	C	C	C
S	C22686T	S375F	T	T	T
S	G22813T	K417N	NR^*a*^	NR	T
S	T22882G	N440K	NR	NR	G
S	G22898A	G446S	NR	NR	A
S	G22992A	S477N	A	A	A
S	C22995A	T478K	A	A	A
S	A23013C	E484A	C	C	C
S	A23040G	Q493R	G	G	G
S	G23048A	G496S	A	A	A
S	A23055G	Q498R	G	G	G
S	A23063T	N501Y	T	T	T
S	T23075C	Y505H	C	C	C
S	C23202A	T547K	A	A	A
S	A23403G	D614G	G	G	G
S	C23525T	H655Y	T	T	T
S	T23599G	N679K	G	G	G
S	C23604A	P681H	A	A	A
S	C23854A	N764K	A	A	NR
S	G23948T	D796Y	T	T	NR
S	C24130A	N856K	A	A	A
S	A24424T	Q954H	T	T	T
S	T24469A	N969K	A	A	A
S	C24503T	L981F	T	T	T
E	C26270T	T9I	T	T	NA
M	A26530G	D3G	G	G	NA
M	C26577G	Q19E	G	G	NA
M	G26709A	A63T	A	A	NA
N	C28311T	P13L	T	T	NA
N	28362_28370del	del31/33	G-[GAGAACGCA]	G-[GAGAACGCA]	NA
N	G28881A, G28882A, G28883C	RG203KR	AAC	AAC	NA

^*a*^ NR, no result.

^*b*^ NA, not available.

### Suspected Omicron variant sample

Three milliliter of nasopharyngeal exudate obtained from a patient arriving from South Africa on 28–29 November 2021 was available.

### RNA extraction and purification

RNA extraction for diagnosis was performed using the KingFisher instrument (ThermoFisher Scientific, Waltham, MA, USA), starting from 300 µL of nasopharyngeal sample and obtaining 50 µL of eluted volume. For variant identification assays, 900 µL of nasopharyngeal sample was extracted in three 300 µL batches, using the EasyMag (Biomerieux, Marcy-l'Etoile, France) automated extraction system, and obtaining 150 µL of RNA.

### Diagnostic test

Diagnosis was made by RT-PCR using the Taq-Path RT-PCR enzyme (ThermoFisher, Waltham, MA, USA), which simultaneously analyzes three genes (N, S, and ORF1). The S-gene dropout was used as a proxy for 69/70 deletion in Omicron.

### Identification of mutations by RT-PCR melting curve analysis

Using commercial VirSNiP SARS-CoV-2 Spike probes (TIB Molbiol, Berlin, Germany) available in our laboratory on November 29, RT-PCR melting curve analyses were performed to detect the presence of different mutations (K417N, E484K, P681R, and L452R). The Lightcycler multiplex RNA Virus Master kit (Roche Diagnostic, Basel, Switzerland) with reverse transcriptase activity was used, following the manufacturer’s instructions. Thermocycler conditions: reverse transcription 53°C/10 min; denaturation: 95°C/2 min; amplification 40 cycles (95°C/3 s, 60°C/3 s); 40°C /30 s; melting: 95°C /30 s, ramp rate 4.4°C /s; 30°C (for E484 assay) and 40°C /2 s, ramp rate 0.2°C /s; and 75°C continuous fluorescence acquisition 3 /s.

### Sanger sequencing

Nine primer pairs from the Artic_nCov-2019_V3 panel set (https://artic.network/ncov-2019) covering the 33 signature Omicron mutations in the S-gene were selected [primers 72 (21658–22038), 73 (21961–22346), 75 (22516–22903), 76 (22797–23214), 77 (23122–23522), 78 (23443–23847), 79 (23789–24169), 80 (24078–24467), and 81 (24391–24789)]. Reverse transcription was performed in duplicate, using the LunaScript RT Supermix kit (NEB, Ipswich, MA, USA) to ensure sufficient volume for all nine reactions. For amplification, AmpliTaq Gold DNA Polymerase (Applied Biosystems, Waltham, MA, USA) was used in a final volume of 25 µL containing 2.5 mM of MgCl_2_, 200 µM of dNTPs, and 0.5 µM of primers, according to the manufacturer’s instructions. Thermocycler conditions were as follows: 95°C/10 min; 30 cycles: 95°C /1 min, 60°C/1 min, 72°C/1 min; 72°C/10 min. PCR products were sequenced on an ABI 3130 × 1 DNA analyzer, following the protocol supplied by the manufacturer. Sequencing data were analyzed with FinchTV software, version 1.4.0.

### Whole-genome sequencing with Oxford Nanopore Technology (ONT)

Reverse transcription was performed with the LunaScript RT SuperMix kit, following the manufacturer’s instructions. For genome amplification, the Q5 Hot Start DNA Polymerase enzyme (NEB, Ipswich, MA, USA) was used, with primers from the Artic_nCov-2019_V4 panel (Integrated DNA Technologies, Newark, NJ, USA; https://artic.network/ncov-2019) and performed in two reactions corresponding to two primer pools. PCR conditions: 98°C/30 s; 35 cycles: 98°C/15 s and 65°C/5 min. Amplification was carried out in duplicate to obtain a larger amount of DNA. After cleaning with CleanNGS (CleanNA, The Netherlands), 30 µL was eluted and a concentration of 132 ng/µL was obtained. For library preparation, the Rapid Barcoding kit (SQK-RBK110.96; Oxford Nanopore technologies) was used. Briefly, 5 µL of the mixture from the two amplification pools was added to 2.5 µL of barcode and 2.5 µL of water, again in duplicate, keeping the same barcode in all four mixes. These were incubated at 30°C/2 min and 80°C/2 min and the four samples were pooled, cleaned as indicated in the protocol, using AMPure XP (Beckman Coulter, Brea, CA, USA), and quantified using a Quantus fluorometer. The concentration was 89 ng/µL, and 9 µL was loaded onto a R9.4.1 flow cell to reach the 800 ng required for optimal sequencing throughput (PCR tiling of SARS-CoV-2 with the rapid virus barcoding protocol, ONT). It was run on the MinION device and stopped at 33 min, at which point the analysis pipeline was launched to assign lineage and mutations identified in the genome.

### Whole-genome sequencing with Illumina technology

The suspected sample was included in our laboratory’s weekly run of 96 samples. The protocol has been described elsewhere (12). Briefly, reverse transcription was performed using the LunaScript RT SuperMix kit. Whole-genome amplification of the genome was performed using the Artic_nCov-2019_V4 panel of primers (IDT) (https://artic.network/ncov-2019) and Q5 Hot Start DNA polymerase. Libraries were prepared using the Nextera DNA prep kit (Illumina, San Diego, CA, USA), following the manufacturer’s instructions, and quantified with a Quantus fluorometer before pooling at equimolar concentrations (4 nM). The 96 libraries were then sequenced using the MiSeq Reagent kit V2 (2 × 150) on a MiSeq device (Illumina).

### Bioinformatic analyses

The sequences obtained were analyzed using in-house pipelines deposited in GitHub:

Sequence obtained using Oxford Nanopore Technology: https://github.com/MG-IiSGM/virION

Sequence obtained using Illumina Technology: https://github.com/MG-IiSGM/covid_multianalysis

For lineage assignment, the pangolin tool was used (v.3.1.16).

## RESULTS AND DISCUSSION

This study was triggered by an alert on the night of 28–29 November 2021 of the possible entry of an Omicron variant after a SARS-CoV-2 RT-PCR-positive specimen (Ct 15) was detected in a traveler arriving in Spain from South Africa and diagnosed after the sample showed S-gene target failure (SGTF) due to the 69/70 deletion. At the epidemiological moment of this first Omicron alert in Spain, the Delta variant was predominant. The first line of testing for suspicion of Omicron was based on identification of SGTF, which is absent in Delta but present in Omicron. The aim of our study was to use the alert of the first indication of Omicron in Spain as an opportunity to perform a rapid investigation of a single high-priority sample in a real-life public health alert using four different methodological strategies tailored to offer different response times and levels of accuracy for laboratories with different resource capabilities.

### Identification of potential VOC candidates or accurate confirmation on the day of diagnosis

#### Strategy 1: Targeted RT-PCR screening for informative mutations

**Fig 1:**
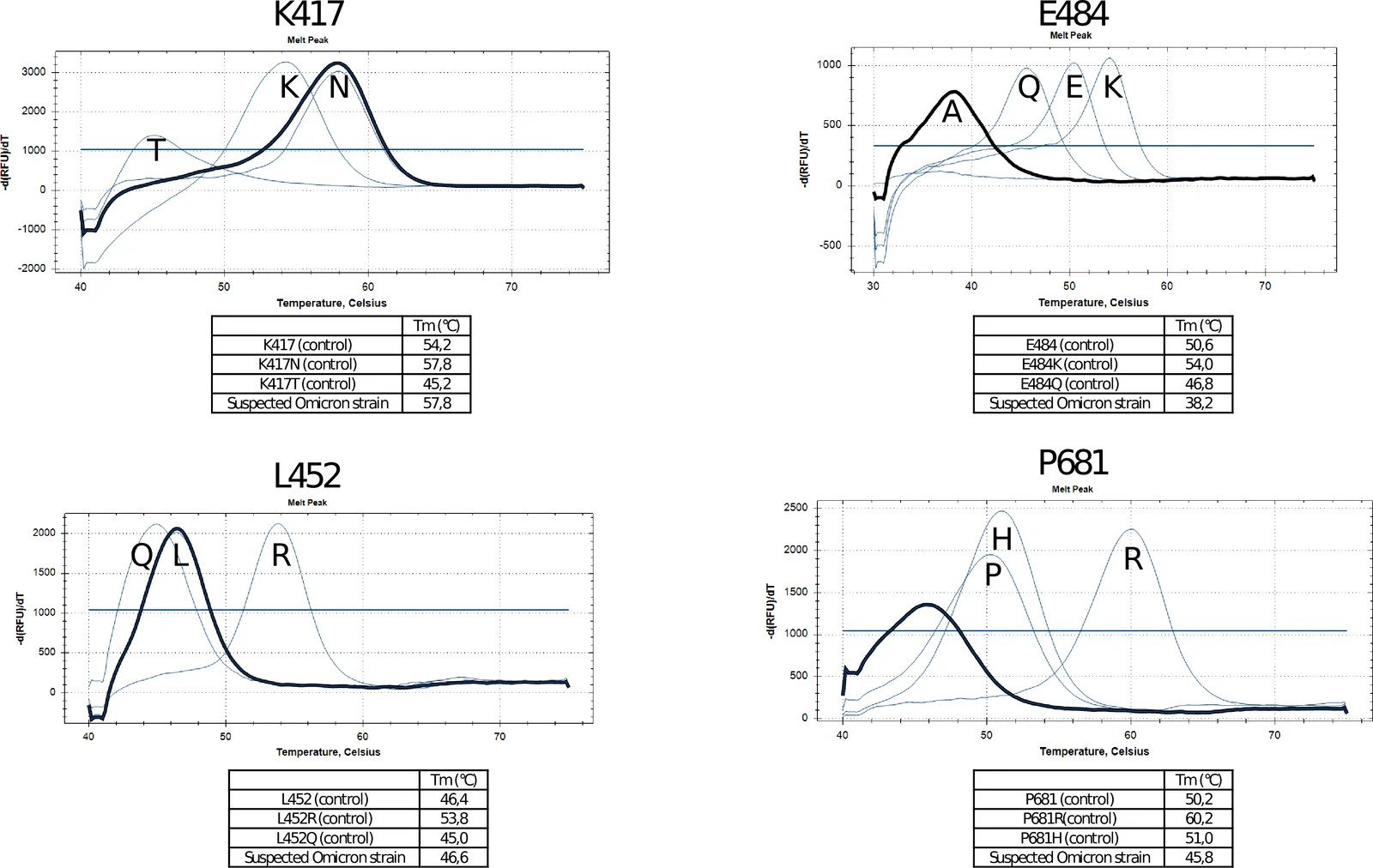
Representation of melting curves and melting temperature values (Tm, see tabular details below the graphs) for analysis of the different mutations analyzed. The thin line shows the controls for each mutation; the thick line shows the result obtained for the suspected Omicron variant sample. Letters indicate the amino acid resulting from each mutation.

The aim of this approach was to obtain reasonably accurate pre-assignment of variants, by RT-PCR screening for the presence/absence of a selected set of signature mutations. We applied our test using probes targeting codons 417, 484, and 452 in the Omicron alert sample. The melting temperature values obtained and deviations from the reference codon values were consistent with the presence of two Omicron signature mutations: K417N (no deviation), E484A (−15.8°C), and the absence of the Delta marker L452 (−7.2°C) (Fig. 1); Delta was the majority variant in our population at that time, in 100% of all new cases in the 15 days prior to this Omicron alert. Interestingly, with probe 681, the Tm deviation value was 5.2°C lower than the one expected for the P681H Omicron signature mutation, as shown in Fig. 1. As Omicron harbors a mutation at a neighboring N679K position, it may be that the presence of the second mutation enhances impairment of the 681 melt temperature, meaning that this melting behavior could act as a separate thermotype for the tandem mutations P681H/N679K and represent a robust signature for Omicron.

**Fig 2:**
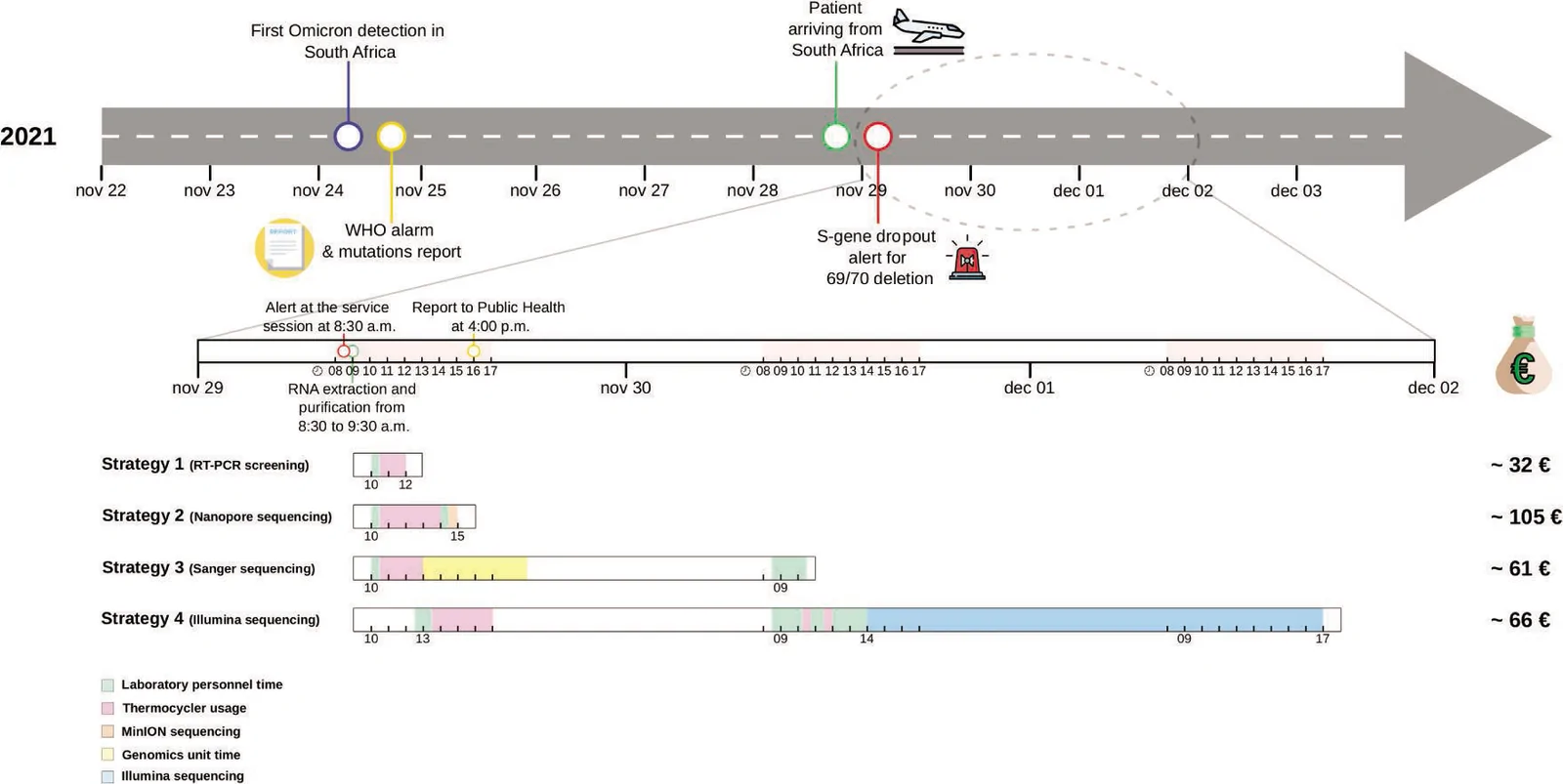
Graphical representation of the key moments of intervention in response to the arrival of the new Omicron variant, with a zoomed-in timeline and the cost of each individual strategy employed. Green represents laboratory personnel time; pink represents thermocycler use; blue represents Illumina sequencing; orange represents MinION sequencing, and yellow represents Genomics Unit time.

Using this strategy, we extracted the information from the four informative sites in just 2 h (Fig. 2). The combination of the four alleles identified is highly predictive of Omicron, as all sequences deposited in GISAID between October 1 and November 30, 2021 with this set of alleles (1542 sequences) belonged to the Omicron variant. Finally, using only the probe that simultaneously identifies the tandem mutations (P681H + N679K), we were able to assign the Omicron VOC with virtually 100% certainty, since only 0.14% of sequences deposited during that period (October–November 2021) were non-Omicron strains.

This strategy demonstrates the versatility of using single probes in diagnostic laboratories to pre-screen SARS-CoV-2 variants. As Omicron shares several of the mutations that were also selected as markers for previous VOCs, we were able to reutilize several probes used prior to Omicron to pre-screen for VOCs. This flexibility is not possible with standard commercial kits, which premix combinations of probes for each specific VOC. Applying this approach, the approximate cost was 32.20 € (Fig. 2).

#### Strategy 2: Ultrarapid whole-genome nanopore sequencing using Oxford Nanopore technology

As our goal was to provide the fastest possible confirmation of genomic response once the Omicron alert had been triggered, nanopore sequencing using MinION device was selected because it offers greater flexibility. Our aim was to use the entire sequencing capacity of the flow cell on a single specimen in order to speed up results.

Once the flow cell was loaded,

78.96 Mb of data were acquired after only 33 min, enabling lineage of the sample to be assigned as B.1.1.529, Omicron. Sequencing indicators were excellent: 849.73X mean depth, 1.27% of unmapped genome sequence reads, and 97% of the genome with >30X coverage. Fifty-one SNPs and seven indels were called. The sequence was found to have 47 of the 50 non-synonymous mutations and indels established for Omicron at that time, 30 of which were located in the S-gene (Table 1). We confirmed the tandem mutation, P681H + N679K, which validated our proposal to use the signature Omicron thermotype identified in the RT-PCR-based approach. Three SNPs in the S-gene (G22813T-K417N; T22882G-N440K; G22898A-G446S) were not detected. This was not due to the limitations of our approach, but to the defective amplification reported with ARTIC primer set version 4.0 due to the accumulation of mutations in that region in Omicron. The estimated cost of applying this sequencing strategy was 105.38 € (Fig. 2).

As a result of these two one-day response strategies, the first Omicron variant in Spain was pre-assigned just 2 h after receipt of RNA from the suspected case, and genomically confirmed 3 h later, allowing for timely reporting to the Public Health authorities.

### Identification of potential VOC candidates or accurate confirmation after the day of diagnosis

#### Strategy 3: High-confidence variant assignment using Sanger sequencing

Seeking an alternative in settings unable to perform NGS, we designed a strategy to increase the confidence of Omicron assignment. This third approach was based on extensive characterization of mutations by Sanger sequencing of pre-selected regions in the S-gene.

Optimal quality sequences were obtained for all cases, except for amplicons 73 and 79, which meant that four mutations (del212/212, 215EPEins, N764K, and D796Y) were missed. Twenty-nine of the 33 Omicron variant mutations in the S-gene were finally identified (Table 1). The sequencing unit at our institution returned the results within 6 h of receipt of the amplicons. The approximate cost was 61.17 € (Fig. 2).

This third approach enabled us to give an Omicron assignment with high confidence within a fairly reasonable time frame: the day after diagnosis. This strategy offers virtually definitive confirmation of the Omicron variant, since no non-Omicron sequences deposited in GISAID since the beginning of the pandemic carry the 29 alleles identified.

#### Strategy 4: Whole-genome sequencing by Illumina technology

Finally, the alert sample was included in the routine weekly run of 96 specimens using Illumina technology. In this context, library preparation required 2 working days and the sequencing run took 28 h. The in-house pipeline applied to obtain the lineage took 1 min per sample and assigned the variant in question to Omicron (Fig. 2). As in our nanopore sequencing-based strategy, only 47 of the 50 mutations described for this variant were detected due to impaired amplification using ARTIC V4.0 primers (Table 1). The sequence quality parameters reached a mean coverage of 1528.84X, leaving only 0.62% of the genome unmapped and 97% genome coverage above 30X. All 47 mutations identified by the nanopore sequencing strategy were confirmed using the Illumina strategy. The estimated cost of a sample in this context was 65.97 € (Fig. 2).

The great advantage of the effort devoted to the diagnosis and study of SARS-CoV-2 in the last 2 years is that most of the molecular biology and genomic tools developed can be readily recycled for the identification of new emerging variants. This in turn makes the materials available to SARS_CoV_2 diagnostic and surveillance laboratories so that they can be ready to optimize rapid identification of new emerging cases.

The main thinking behind our set of methodological proposals was to recognize that not all laboratories have the same technical and human resources available to deal with public health alerts arising from the suspected introduction of a new VOC. Our intention was to explore four alternative strategies offering different levels of accuracy and response times: between 2 h and 3 days. These diverse proposals, tailored to the varying capabilities of different laboratories, offer a range of options to provide the fastest and most accurate response possible to the competent authorities, enabling them to initiate interventions related to patient isolation and contact surveillance.

## Data Availability

The sequences were deposited on the GISAID.org platform: EPI_ISL_6851526 (ONT) and EPI_ISL_14917977 (Illumina).
